# Alcohol and Dopamine

**Published:** 1997

**Authors:** Gaetano Di Chiara

**Affiliations:** Gaetano Di Chiara, M.D., is chairman of the Department of Toxicology, University of Cagliari, Italy

**Keywords:** dopamine, dopaminergic receptors, cell signaling, neurotransmission, reinforcement, motivation, neurotransmitters, nucleus accumbens, brain, neuron, sensory stimuli, AOD craving, AOD dependence, neurobiological theory, literature review

## Abstract

Dopamine is a neuromodulator that is used by neurons in several brain regions involved in motivation and reinforcement, most importantly the nucleus accumbens (NAc). Dopamine alters the sensitivity of its target neurons to other neurotransmitters, particularly glutamate. In addition, dopamine can affect the neurotransmitter release by the target neurons. Dopamine-containing neurons in the NAc are activated by motivational stimuli, which encourage a person to perform or repeat a behavior. Even low alcohol doses can increase dopamine release in part of the NAc. This dopamine release may contribute to the rewarding effects of alcohol and may thereby play a role in promoting alcohol consumption. In contrast to other stimuli, alcohol-related stimuli maintain their motivational significance even after repeated alcohol administration, which may contribute to the craving for alcohol observed in alcoholics.

Many substances that relay signals among neurons (i.e., neurotransmitters) are affected by alcohol. Among these, dopamine has received special attention, because several studies have found that alcohol stimulates the activity of a subset of dopamine-releasing neurons and thus enhances dopamine-mediated (i.e., dopaminergic^1^) signal transmission in a discrete brain area called the nucleus accumbens (NAc) (Di Chiara and Imperato 1985; Imperato and Di Chiara 1986; Gessa et al. 1985). Alcohol shares this property with most substances of abuse (Di Chiara and Imperato 1988), including nicotine, marijuana, heroin, and cocaine (Pontieri et al. 1995, 1996; Tanda et al. 1997). These observations have stimulated many studies on dopamine’s role in alcohol abuse and dependence, also with the intent of finding new pharmacological approaches to alcoholism treatment. This review summarizes some of the characteristics of dopaminergic signal transmission as well as dopamine’s potential role in alcohol reinforcement.

## Dopamine Production and Distribution in the Brain

Dopaminergic neurons produce dopamine from the dietary amino acid tyrosine. The neurons then store the dopamine in small compartments (i.e., vesicles) in the terminals of their axons. When the dopaminergic neurons are activated, the resulting change in the electrical charges on both sides of the cell membrane (i.e., depolarization) induces dopamine release into the gap separating the neurons (i.e., the synaptic cleft) through a process called exocytosis. (For more information on the processes involved in nerve signal transmission within and among neurons, see the article “The Principles of Nerve Cell Communication,” pp. 107–108.) To terminate the signaling process, the neurons recapture dopamine through a specific carrier system located on the cell membrane.

### Distribution of Dopaminergic Neurons

**Figure 1:**
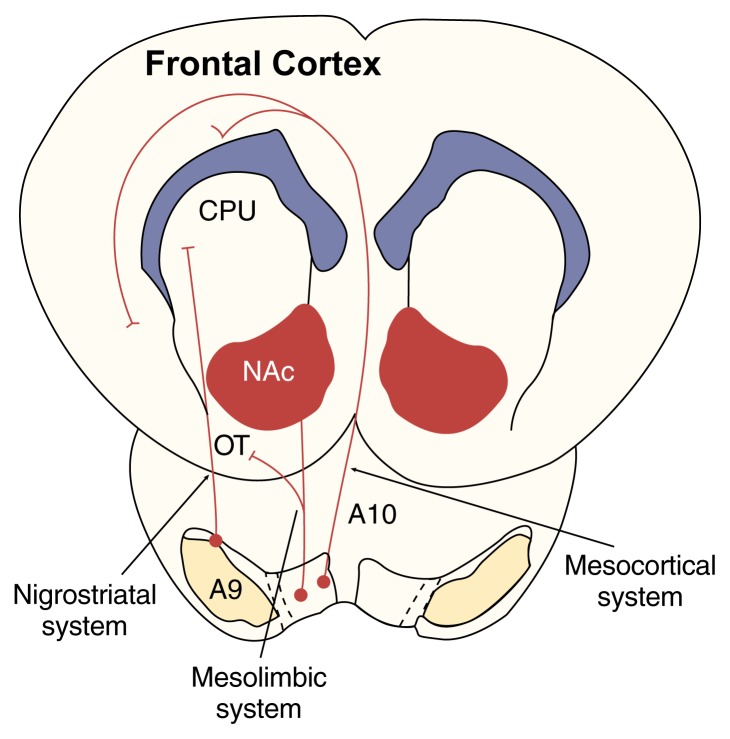
Schematic representation of the major dopaminergic systems (viewed from the top of the head). The nigrostriatal system originates in the A9 cell group and extends to the dorsal striatum, which includes the caudate nucleus and putamen (CPU). The mesolimbic system originates primarily in the A10 cell group and extends to the ventral striatum, which includes the nucleus accumbens (NAc) and the olfactory tubercle (OT). The mesocortical system also originates primarily in the A10 cell group and affects various regions of the cerebral cortex. SOURCE: Adapted with permission from Di Chiara, G. In vivo brain dialysis of neurotransmitters. *Trends in Pharmacological Sciences* 11:116–121, 1990.

Dopaminergic neurons originate in three cell groups located in the brain stem. These cell groups are labeled A8, A9, and A10 and correspond to brain regions called the retrorubral field (A8), the substantia nigra pars compacta (A9), and the ventral tegmental area (VTA) (A10). From these cell groups, the axons of dopaminergic neurons extend to discrete regions of the forebrain, forming the following three neuronal systems (figure 1):

- The *nigrostriatal system* comprises dopaminergic neurons that originate in the A9 group and terminate in a region called the dorsal striatum. This region, which includes the caudate nucleus and the putamen, is involved in learning to automatically execute complex movements triggered by a voluntary command (e.g., driving a car). The degeneration of dopaminergic neurons in the dorsal striatum causes the motor disturbances that occur in Parkinson’s disease.
- A second circuit, the *mesolimbic system*, originates in the A10 and part of the A9 groups. These neurons terminate in the ventral striatum, which includes the NAc and the olfactory tubercle, the septum, the central amygdala, and the bed nucleus of the stria terminalis (Ungerstedt 1971). The ventral striatum plays a role in the learning and performing of certain behaviors in response to incentive stimuli (i.e., motivation).
- A third group of dopaminergic neurons originates in the A9 and A10 groups and terminates in various regions of the cerebral cortex that are involved in attention and short-term memory, forming the *mesocortical system* (Thierry et al. 1973).

This rather specific distribution pattern of dopaminergic neurons contrasts with other related neurotransmitter systems (e.g., serotonin or noradrenaline), which affect most regions of the forebrain.

**Figure 2:**
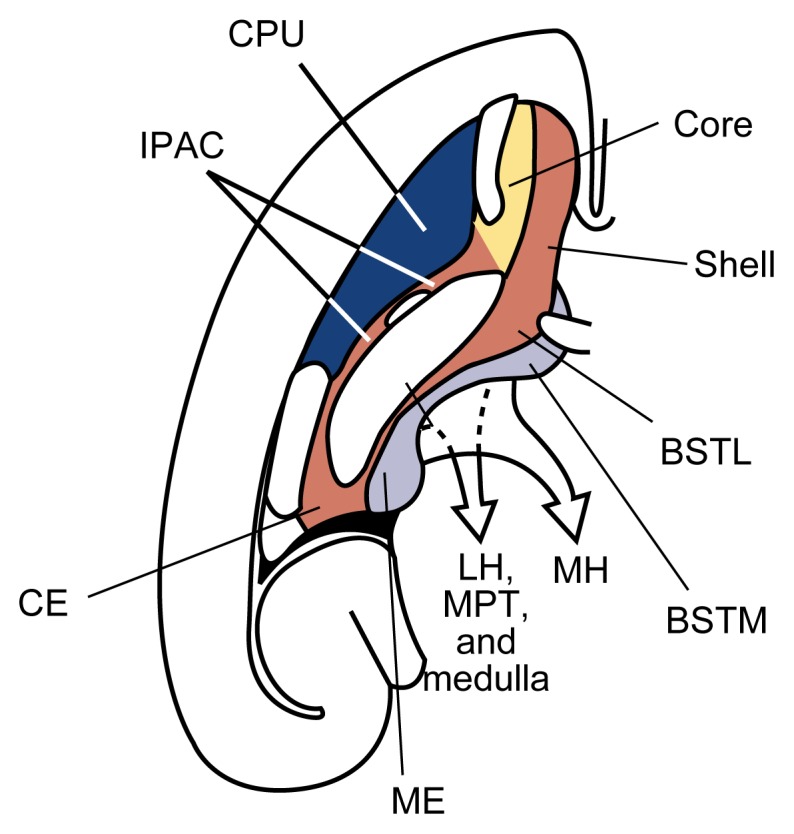
Key areas of the brain (e.g., striatum and extended amygdala) with dopaminergic transition (viewed in a cross-section of the left hemisphere of the brain). The striatum includes the caudate nucleus and putamen (CPU) (dark blue) as well as the nucleus accumbens core (yellow) and shell (red). The extended amygdala is distinguished into a central division (red) and a medial division (light blue). The central division includes the nucleus accumbens shell, the lateral part of the bed nucleus of stria terminalis (BSTL), the central amygdala (CE), and other neuronal groups bridging these areas (IPAC). From these structures, neurons extend to the lateral hypothalamus (LH), visceral nuclei in the brain stem (MPT), and medulla. The medial division of the extended amygdala includes the medial part of the bed nucleus of stria terminalis (BSTM), the medial amygdala (ME), and other associated neuronal groups. Neurons originating in the medial division extend to the medial hypothalamus (MH). SOURCE: Adapted with permission from Heimer et al. 1997.

Among the brain areas affected by dopaminergic neurons, the NAc plays a pivotal role. The NAc can be divided into a “shell” region and a “core” region (Heimer et al. 1997) (figure 2). The shell is part of a complex of brain structures called the extended amygdala, which is involved in integrating emotions to elicit appropriate motor, autonomic^2^ (i.e., visceral), and hormonal responses. The NAc core, in contrast, belongs to a group of brain areas called the striato-pallidal system, which plays a role in the integration of motor responses. The NAc interacts with three major brain systems: It receives motivationally relevant information from a brain system called the septo-hippocampal system, which is involved in learning and memory. The NAc then funnels this information to other parts of the extended amygdala and the striato-pallidal system to elicit voluntary motor responses, visceral motor responses, and hormonal responses.

Dopaminergic neurons reach not only the NAc, but also other areas of the extended amygdala as well as parts of the septo-hippocampal system. Consequently, dopamine acts at multiple sites to control the integration of biologically relevant information that determines motivated responding.

## Cellular Actions of Dopamine

To affect its target cells, dopamine interacts with specific docking molecules (i.e., receptors) on the target-cell surface. Dopamine receptor subtypes fall into two families: the D_1_-like family, which includes the receptor subtypes D_1_ and D_5_, and the D_2_-like family, which includes the receptor subtypes D_2_, D_3_, and D_4_. When dopamine interacts with these receptors, they activate certain proteins within the cell called G proteins. These proteins, in turn, can affect the activity of channels in the cell membrane that allow the flow of charged particles (i.e., ions) into and out of the cell. Changes in the activity of these ion channels make it either easier or more difficult to excite the cell. In addition, G proteins regulate the production of small signaling molecules (i.e., second messengers) within the cell. (For more information on G proteins and second messengers, see the article by Dohrman and colleagues, pp. 136–143.)

Because dopamine does not affect the activity of ion channels directly and therefore is unable to excite or inhibit its target cells, it often is not considered a neurotransmitter but is called a neuromodulator (Kitai and Surmeier 1993; Di Chiara et al. 1994). Thus, dopamine modulates the efficacy of signal transmission mediated by other neurotransmitters. Dopamine exerts its effects through two distinct mechanisms (Di Chiara 1995). First, dopamine alters the sensitivity with which dopamine-receptive neurons respond to stimulation by classical neurotransmitters, particularly glutamate.^3^ This mechanism is referred to as the phasic-synaptic mode of dopaminergic signal transmission. Second, dopamine can modulate the efficacy with which electrical impulses generated in dopaminergic or nondopaminergic neurons result in neurotransmitter release from the nerve terminals of these signal-emitting (i.e., pre-synaptic) cells. This presynaptic influence is part of the tonic-nonsynaptic mode of dopaminergic signal transmission.

### Dopamine’s Phasic-Synaptic Actions

To modulate the responsiveness of neighboring neurons to glutamate, dopamine modifies the function of ion channels in the membrane of the signal-receiving (i.e., postsynaptic) neuron. The activity of some of these ion channels (i.e., whether they are open or closed) depends on the voltage difference, or potential, between the inside and the outside of the cell membrane adjacent to these channels. These channels therefore are called voltage-dependent channels. Through its effects on G proteins, dopamine indirectly modifies the sensitivity with which voltage-dependent channels respond to changes in the membrane potential that occur when glutamate binds to its receptors, which also act as ion channels (i.e., receptor-operated channels).

Dopamine’s effects on neuronal function depend on the specific dopamine-receptor subtype that is activated on the postsynaptic cell. For example, different subpopulations of neurons in the striatum carry different dopamine receptors on their surfaces (Le Moine et al. 1990, 1991; Gerfen 1992). Dopamine binding to D_1_ receptors enhances the excitatory effects that result from glutamate’s interaction with a specific glutamate receptor subtype (i.e., the NMDA receptor^4^). Conversely, activation of D_2_ receptors inhibits the effects induced by glutamate’s binding to another glutamate-receptor subtype (i.e., the AMPA receptor^5^) (Cepeda et al. 1993). (For more information on glutamate receptor subtypes, see the article by Gonzales and Jaworski, pp. 120–127.) Consequently, dopamine can facilitate or inhibit excitatory neurotransmission, depending on the dopamine-receptor subtype activated. Moreover, even with the same receptor affected, dopamine’s effects can vary, depending on the potential of the membrane where dopamine receptors are activated (Kitai and Surmeier 1993).

### Dopamine’s Tonic-Nonsynaptic Actions

Both dopaminergic and nondopaminergic neurons also carry dopamine receptors that are located on the nerve terminals outside the synapse (i.e., are extrasynaptic). Dopamine that has been released from a nerve terminal into the synaptic cleft can travel out of the synapse into the fluid surrounding the neurons and activate these extrasynaptic receptors. Through this mechanism, dopamine modulates the neurotransmitter release that is induced by cellular excitation (i.e., neurotransmitter secretion). For example, activation of some extrasynaptic D_2_-family receptors can inhibit the release of dopamine itself, thereby reducing dopaminergic signal transmission.

Activation of some extrasynaptic receptors of the D_1_ and D_2_ families also modulates the release of other neurotransmitters (e.g., acetylcholine, glutamate, and gamma-aminobutyric acid) by nondopaminergic neurons (Starke 1981; Chesselet 1984). Thus, dopamine-mediated activation of extrasynaptic D_1_ receptors enhances, and activation of extrasynaptic D_2_ receptors reduces, the release of these neurotransmitters. As mentioned previously, D_1_- and D_2_-family receptors are not evenly distributed among dopaminergic cells (e.g., in the striatum). Accordingly, dopamine’s tonic-nonsynaptic actions could modulate the flow of information across the striatum in the following ways:

- By stimulating D_2_-family receptors, which reduce neurotransmitter release, dopamine could reduce signals that represent “background noise,” thereby ultimately sharpening the transmission of information across the striatum.
- By stimulating D_1_-family receptors, which enhance neurotransmitter release, dopamine could facilitate the transmission of information from the striatum to other brain areas (Spano et al. 1977; Martin and Waszczak 1994).

## Dopamine’s Role in Behavior

A large body of evidence indicates that dopamine plays an important role in motivation and reinforcement^6^ (Wise 1982; Robbins et al. 1989; Di Chiara 1995). The precise role of dopamine in behavior depends on three factors. These factors include (1) the type of stimuli that activate dopaminergic neurons, (2) the specific brain area(s) affected by dopamine, and (3) the mode of dopaminergic neurotransmission (i.e., whether phasic-synaptic or tonic-nonsynaptic).

Dopaminergic neurons are activated by stimuli that encourage a person or animal to perform or repeat a certain behavior (i.e., motivational stimuli). These stimuli converge in the A8, A9, and A10 dopaminergic cell groups. From there, the information is passed on to the various brain areas where dopaminergic neurons terminate. Consequently, through the activation of dopaminergic neurons, motivational stimuli can influence the activity of various parts of the brain that might serve different behavioral functions. This mechanism may be one reason underlying the wide range of dopamine’s roles in behavior.

The diversity of dopamine’s roles also derives from the fact that dopaminergic neurons belonging to different systems (e.g., the nigrostriatal, mesolimbic, and mesocortical systems) respond to different kinds of motivational stimuli. Two kinds of motivational stimuli exist: appetitive and con-summatory. Appetitive stimuli serve to attract an individual to a reward (e.g., food) so that the individual can attain it. Consummatory stimuli serve to maintain the individual’s contact with the reward so that the individual can use its biological properties (e.g., the caloric content of food). Some dopaminergic neurons (e.g., mesocortical neurons) are activated by both appetitive stimuli (e.g., the smell of a food or the sight of a specific bowl in which the food is always served) and consummatory stimuli (e.g., the taste of a highly palatable food). Conversely, other dopaminergic neurons (e.g., mesolimbic neurons) are activated only by consummatory stimuli (Bassareo and Di Chiara 1997).

The different modes of dopaminergic signal transmission also might serve different functions in controlling behavior. For example, dopamine’s phasic-synaptic actions in the NAc shell, which occur in response to natural stimuli such as food, may play a role in motivational learning (Bassareo and Di Chiara 1997). This term refers to the process by which a neutral stimulus that is consistently associated with a reward acquires the ability to elicit motivated behavioral responses. For example, a light that flashes each time an animal is fed becomes a motivational stimulus that induces the animal to search for food. Similarly, stimuli such as the bottle of a specific brand of beer or the description of the taste of an alcoholic beverage may become motivational stimuli for alcohol consumption through dopamine’s phasic-synaptic actions (see below). Dopamine’s tonic-nonsynaptic actions, in contrast, are involved in the motor expression of motivated behaviors.

**Motivation and Reinforcement**

When discussing the consequences of alcohol’s actions on the brain, researchers frequently use terms such as motivation, reinforcement, incentives, and reward. Below are general definitions of these terms.

*Incentives* are stimuli that induce behaviors with the goal of obtaining a reward (e.g., food). Thus, incentives promote responses (e.g., the search for, approach to, and contact with the reward) necessary for obtaining the reward’s biologically relevant properties (e.g., calories). These responses also can include emotions. Incentives themselves have no primary biological properties that could induce motivated behavior. Therefore, incentives obtain motivational properties by being predictably associated with primary biological stimuli.

*Motivation —* a process by which stimuli (e.g., the smell of food) come to trigger responses to obtain a reward (e.g., a palatable food) or to avoid a punishment (e.g., a painful electrical shock) — generally serves to maintain bodily functioning and ensure survival.

*Motivational learning* is the mechanism by which secondary stimuli acquire the ability to elicit behavioral responses. Two types of motivational learning exist: incentive learning and habit learning. In incentive learning, the stimulus acquires the ability to elicit motor as well as emotional responses. In habit learning, the stimulus elicits only physical (i.e., somatic) responses but is devoid of an emotional content.

*Reinforcement* exists in two types: pavlovian, in which the predictable association of a reward (e.g., food) with a neutral stimulus (e.g., a flashing light) strengthens the ability of that stimulus to elicit a response, and operant, in which the stimulus acts as a reinforcer by eliciting a response that enables the subject to obtain (positive reinforcement) or to avoid (negative reinforcement) the same stimulus in the future.

*Rewards* are the goals of motivated behavior. A reward (e.g., food) usually is a complex stimulus having primary (e.g., calories) as well as secondary (e.g., taste and smell) motivational properties.

*Stimuli* that elicit behavioral responses fall into two categories: primary and secondary. Primary stimuli elicit responses or maintain behaviors without requiring the presence of additional stimuli or prior learning processes. Secondary stimuli depend on primary stimuli to induce a response.

However, some food-related stimuli (e.g., taste) that activate phasic-synaptic dopaminergic signal transmission in the NAc shell rapidly undergo a form of tolerance (i.e., habituation) (Bassareo and Di Chiara 1997). For example, rats receiving a palatable food for the first time exhibited significant dopaminergic signal transmission in the NAc shell. A second feeding session that took place within 1 day of the first feeding session, however, induced no or only weak dopaminergic signal transmission. Only about 5 days after the first feeding session did the animals recover the full dopaminergic response to this stimulus. As discussed later in this article, however, alcohol does not induce a comparable habituation.

## Alcohol’s Effect on the Dopamine System

Dopaminergic neurons that relay information to the NAc shell are extremely sensitive to alcohol. For example, in studies performed in rats, alcohol injected into the blood in amounts as low as 2 to 4 milligrams per kilogram of body weight increased dopamine release in the NAc shell and maintained chronic alcohol self-administration (Lyness and Smith 1992). In rats, oral alcohol uptake also stimulates dopamine release in the NAc (Weiss et al. 1995). To achieve the same effect, however, this administration route requires higher alcohol doses than does alcohol injection directly into the blood.

The alcohol-induced stimulation of dopamine release in the NAc may require the activity of another category of neuromodulators, endogenous opioid peptides. (For more information on endogenous opioid peptides, see the article by Froehlich, pp. 132–136.) This hypothesis is supported by observations that chemicals that inhibit the actions of endogenous opioid peptides (i.e., opioid peptide antagonists) prevent alcohol’s effects on dopamine release. Opioid peptide antagonists act primarily on a brain area where dopaminergic neurons that extend to the NAc originate. These observations indicate that alcohol stimulates the activity of endogenous opioid peptides, leading indirectly to the activation of dopaminergic neurons. Opioid peptide antagonists would interfere with this process, thereby reducing dopamine release.

## Alcohol’s Actions as a Reinforcer: Dopamine’s Role

Although numerous studies have attempted to clarify dopamine’s role in alcohol reinforcement by manipulating dopaminergic signal transmission, these investigations do not allow any firm conclusions (for a review, see Di Chiara 1995). The comparison of alcohol’s effects with the effects of conventional reinforcers, such as food, however, provides some clues to dopamine’s role in mediating alcohol reinforcement.

Palatable food activates dopaminergic signal transmission in the NAc shell, for example, by exerting specific sensory (e.g., taste, or gustatory) stimuli. Orally administered alcohol similarly activates taste receptors, thereby increasing dopamine release in the NAc. In contrast to food, however, alcohol also can modify the function of dopaminergic neurons more directly by entering the brain. Accordingly, oral alcohol administration influences dopamine release in the NAc both through its gustatory properties (i.e., as a conventional reinforcer) and through its direct actions on the brain (i.e., as a drug reinforcer). Consistent with this hypothesis, two peaks of dopamine release occur in the NAc. The first peak results from the alcohol-related gustatory stimuli; the second results from alcohol’s actions within the brain. Consequently, alcohol-induced direct activation of dopaminergic signal transmission might reinforce the motivational properties of the gustatory stimuli associated with alcohol. As a result of this mechanism, the alcohol-related gustatory stimuli acquire strong incentive properties (i.e., they become motivational stimuli that induce the drinker to seek even more alcohol). Similarly, appetitive stimuli related to alcohol (e.g., extrinsic stimuli, such as the sight of a certain brand of an alcoholic beverage or the sight of a bar) also acquire incentive properties and promote the search for and consumption of alcohol. Through these complex mechanisms, the alcohol-induced dopamine release activates a secondary reinforcement chain that promotes alcohol consumption.

## Dopamine’s Role in the Development of Alcohol Dependence

Dopamine release in the NAc shell may be instrumental in the development of alcohol dependence. Psychological dependence on alcohol develops because alcohol-related stimuli acquire excessive motivational properties that induce an intense desire to consume alcohol-containing beverages (i.e., craving). As a result of this intense craving, conventional reinforcers (e.g., food, sex, family, job, or hobbies) lose their significance and have only a reduced impact on the drinker’s behavior.

One mechanism that may be responsible for the abnormal significance associated with alcohol-related incentives is the nonadaptive nature of alcohol-induced stimulation of dopaminergic signal transmission in the NAc. As mentioned previously, enhanced dopamine release in the NAc shell induced by conventional reinforcers (e.g., food) rapidly induces habituation, and repeated presentation of related stimuli no longer induces dopamine release. In contrast, no habituation occurs after repeated alcohol consumption. As a result of the persistent dopamine release in the NAc shell in response to alcohol, alcohol-associated stimuli acquire an abnormal emotional and motivational significance that results in excessive control over the drinker’s behavior. This excessive control constitutes the essence of addiction.

## Summary

Dopaminergic signaling plays a pivotal role in the transmission of motivational stimuli. Dopaminergic neurons primarily affect brain areas involved in mediating the rewarding and reinforcing properties of alcohol and other drugs, most prominently the NAc. Alcohol affects dopamine release in the NAc, not only through its associated gustatory stimuli but also through its direct actions on the brain. The abnormal facilitation of motivational learning that results from alcohol-induced stimulation of dopaminergic signal transmission has been hypothesized to constitute the neurobiological basis of alcohol addiction. Through this mechanism, alcohol-associated stimuli acquire the ability to elicit craving and compulsive alcohol consumption.

## Footnotes

1 The term “dopaminergic” refers to both the neurons and the signaling processes that use dopamine. See figure in glossary, pp. 177–179, for more information.

2 Autonomic, or visceral, responses regulate the involuntary bodily functions, such as heart rate, blood pressure, and gastrointestinal activity.

3 Glutamate is the major excitatory neurotransmitter; that is, glutamate stimulates the signal-receiving cell.

4 *N*-methyl-d-aspartate, or NMDA, is a chemical that specifically activates this glutamate-receptor subtype.

5 Aminomethyl propionic acid, or AMPA, is a chemical that specifically activates this glutamate-receptor subtype.

6 For a definition of these and other related terms, see box, p. 112.
